# Neurons ensheathed by perineuronal nets are prone to hyperphosphorylation of tau protein in the hibernating Syrian hamster brain

**DOI:** 10.1038/s41598-025-14942-9

**Published:** 2025-09-01

**Authors:** Valerie Berrouschot, Mandy Sonntag, Katja Reimann, Carsten Jäger, Sven Martin, Max Holzer, Jens T. Stieler, Markus Morawski

**Affiliations:** 1 Paul Flechsig Institute – Centre of Neuropathology and Brain Research, Liebigstr. 19, 04103 Leipzig, Germany; 2 Medizinisch-Experimentelles Zentrum of the Medical Faculty of the University of Leipzig, Liebigstr. 19, 04103 Leipzig, Germany; 3 Innovation Center Computer Assisted Surgery (ICCAS), Semmelweisstr. 14, 04103 Leipzig, Germany; 4 Department of Neurophysics, Max Planck Institute for Human Cognitive and Brain Sciences, Stephanstraße 1a, 04103 Leipzig, Germany

**Keywords:** Cellular neuroscience, Neurochemistry, Immunochemistry, Polysaccharides, Glycobiology, Neuroscience

## Abstract

Perineuronal nets (PNNs) are a specialized form of neuronal extracellular matrix that often ensheath highly active cells, such as parvalbumin-positive (PV+) interneurons. Net-bearing neurons have been shown to be devoid of pathological aggregates of hyperphosphorylated tau protein (p-tau), suggesting they may serve neuroprotective functions. P-tau is a major hallmark of tauopathies like Alzheimer’s disease (AD) but is also naturally present in the brains of hibernating mammals during the torpor phase. However, the relationship between PNNs and p-tau in both pathological and physiological contexts remains unclear. In this study, we examined the association between PNNs, PV, and p-tau in the neocortex of hibernating Syrian hamsters. We observed consistent PNN expression across euthermic and torpid states. Unexpectedly, PNN-enwrapped neurons showed a higher prevalence of p-tau (82%) compared to the overall neuronal population (60%) during torpor. Similarly, PV+ interneurons displayed a high prevalence of p-tau (78%). These findings suggest that PNNs do not prevent tau hyperphosphorylation under physiological conditions like hibernation, in contrast to their potential neuroprotective role in AD pathology. Instead, specific neuronal subsets, such as PNN-enwrapped PV+ interneurons, are likely to exhibit p-tau during torpor. Further analysis of the interplay between p-tau, PNNs, and PV+ interneurons may therefore reveal adaptive strategies for neuronal protection and metabolic regulation, with implications for neurodegeneration and brain metabolic stress.

## Introduction

The extracellular matrix (ECM) constitutes roughly 20% of the brain volume^1,2^, yet it has long been overshadowed by the neuronal-glial perspective that dominated neuroscientific discourse. Although brain-specific ECM was initially described by Camillo Golgi in 1882^3^, it was not until decades later that sufficient interest was sparked in what is now understood to be a specialized form of neuronal ECM: the perineuronal net (PNN). PNNs are primarily composed of negatively charged chondroitin sulfated proteoglycans (CSPGs) of the lectican family, encompassing brevican, versican, neurocan, and the predominant CSPG found in mature cortical neurons, aggrecan^4,5^. The lecticans are attached to a hyaluronic acid (HA) backbone, with hyaluronan and proteoglycan link proteins (HAPLNs) reinforcing the bonds between the two components. Additionally, tenascin-R forms crosslinks between CSPGs to stabilize the overall structure, while HA synthase serves to anchor the complex to the cell surface^6,7^. Leaving gaps for synaptic contacts, the dense meshwork of the PNNs enwraps the cell bodies, axon initial segments, and proximal dendrites of specific neuronal subpopulations within the central nervous system^8–10^. For example, PNNs are closely linked to neurons involved in rapid transmission such as glycinergic output neurons in the medial nucleus of the trapezoid body (MNTB) at the calyx of Held synapse^11–13^ and fast-spiking inhibitory interneurons that express the calcium-binding protein parvalbumin (PV)^14^. Given their polyanionic composition, PNNs have been proposed to serve as a stationary ion exchanger, thereby providing the basis for such neurons to sustain their fast-firing activity by preventing free diffusion of cations^15,16^. Indeed, degradation of CSPGs resulted in increased calcium diffusion^17^ and reduced excitability of fast-spiking neurons^18^ in rodent brain slices, indicating that PNNs may contribute to creating the specifically required microenvironment for these nerve cells. Furthermore, the maturation of PNNs is involved in closing critical periods for experience-dependent plasticity during early postnatal development^19–23^; enzymatic breakdown of PNNs in adult rodents reinstated juvenile-like plasticity in several brain regions, including the visual and auditory cortex^23–25^. Besides their role in restricting neuroplasticity in the context of critical periods, PNNs have also been demonstrated to carry out neuroprotective functions. For instance, net-bearing neurons have been reported to be less vulnerable to oxidative stress^26–29^. This is especially important for fast-spiking inhibitory PV+ interneurons, as their high metabolic demands render them particularly susceptible to damage from redox dysregulation^30–32^. Additionally, PNN-enwrapped human neurons almost never exhibit aggregates of hyperphosphorylated tau protein (p-tau)^33–36^, which are a major pathological hallmark of certain neurodegenerative conditions known as tauopathies^37^, such as Alzheimer’s disease (AD)^38–43^. The microtubule-associated protein tau is known to facilitate the assembly and stabilization of microtubules and, consequently, plays an important role in axonal transport and cytoskeletal remodeling, among others^44–47^. Phosphorylation of tau alters these functions, resulting in the detachment of the protein from the microtubules and its subsequent translocation into the somatodendritic compartment^48–50^. There, it accumulates and forms irreversible pathological aggregates which promote neurodegeneration in the case of AD^51–53^. However, reversible increased tau phosphorylation also occurs under non-pathological conditions, including early brain development^54^, anesthesia^55^, hypothermia^56^, starvation^57^, hibernation^58–60^, and some torpor-like rodent models of non-hibernators^61,62^. The latter represents a remarkable adaptive mechanism by which animals conserve energy in order to survive under extreme conditions when ambient temperature and food availability are reduced. In small mammals, this process is characterized by several bouts of deep hibernation, the so-called torpor, which can last for three to four days, interspersed with brief periods of activity and normothermia^63^. During torpor, the overall reduction in basal metabolic rate to less than 1% compared to normothermic conditions is accompanied by a decrease in heart and respiratory rate, body and brain temperature, and the virtual cessation of neuronal activity in the brain^63–65^. Several hibernating species exhibit hyperphosphorylated but not pathologically aggregated tau protein during torpor^66^. Upon arousal, the protein reverts to its original state without causing any apparent brain damage^67^. Hibernation can be induced in facultative hibernators such as the Syrian hamster (*Mesocricetus auratus*) under controlled laboratory conditions, which renders the Syrian hamster a suitable animal model for investigating the reversible physiological hyperphosphorylation of tau. This provides a unique opportunity to examine whether PNN-ensheathed neurons are protected from potentially pathological p-tau, specifically, or if net-bearing cells are devoid of any p-tau in general. For this purpose, we initially compared immunohistochemically determined PNN-expression in the neocortex between euthermic and torpid animals and subsequently analyzed whether net-bearing neurons were devoid of p-tau during hibernation. Given the pronounced PNN and p-tau expression observed in the neocortex in prior work and the documented silencing of this brain region during torpor, all immunohistochemical analyses were conducted in the motor cortex and primary somatosensory cortex as part of the neocortex. We additionally corroborated the strong interconnection between net-bearing neurons and PV+ inhibitory interneurons in the neocortex of the Syrian hamster and examined the subset of PV+ net-bearing neurons with regard to their correlation with p-tau during torpor. Consequently, this knowledge could offer insight into the shared or distinct mechanisms of pathological and physiological tau phosphorylation, thereby facilitating the differentiation between healthy states and diseases such as tauopathies.

## Materials and methods

### Animals and brain tissue preparation

All experimental procedures were performed in accordance with relevant guidelines. Animal experiments were conducted in accordance with the ARRIVE guidelines and were approved by the Saxonian District Government (TVV06/19). For the present study, a total of six adult male Syrian hamsters (*Mesocricetus auratus*) of at least seven months of age were used that were obtained from Janvier Labs (Le Genest-Saint-Isle, France) and housed at the Medizinisch-Experimentelles Zentrum (MEZ) of the Medical Faculty of the University of Leipzig. Upon arrival at the MEZ, the animals were transferred to a climate chamber, which allowed for the regulation of temperature, relative humidity, and illumination while monitoring their locomotor activity. For the acclimatization period (1–2 weeks), they were maintained under conditions of constant ambient temperature (T_a_) of 22 °C and 50–55% relative humidity, as well as an artificial 12:12 h light:dark cycle (L:D). Food and water were always provided ad libitum. Induction of hibernation was generally performed according to Oklejewicz et al.^68^. Over a period of four weeks, the light dark cycle was incrementally changed to 8:16 h (L:D). T_a_ was then gradually lowered to 5 °C, after which the climate chamber was illuminated exclusively by red light (wavelength ≥ 700 nm), which is imperceptible to hamsters. Locomotor activity was monitored using custom-built infrared detectors mounted above each cage, and the recorded signals were processed using the custom software Hibernate 1.0. The hamsters were categorized as euthermic or torpid based on their activity profile and core body temperature (T_b_). After > 24 h of inactivity, animals were deemed to be in a state of torpor. Animals were considered to be torpid (N = 3) only when they had completed at least one full torpor bout and had been in the torpor state of the subsequent bout for a minimum of 24 h. Additionally, their T_b_ before the start of the euthanasia process had to be below 7 °C. In contrast, animals were considered euthermic (N = 3) if they had never entered the torpor state while being kept under hibernation conditions for at least one week and exhibited a T_b_ between 34 and 36 °C at the time of sacrifice. All animals were deeply anesthetized with an isoflurane overdose (5 vol%) following a sedating ketamine injection. The animals were euthanized by a transcardial perfusion resulting in exsanguination. For perfusion, initially a solution of 0.9% saline and 0.1% heparin was used to flush blood cells out of the vessels, followed by a solution of 4% paraformaldehyde and 0.1% glutaraldehyde in phosphate buffered saline (PBS; 0.1M, pH 7.4) to fix the tissue. The brains were removed and postfixed by immersion in the same fixative overnight. Thereafter, the brains were cryoprotected in a solution of 30% sucrose and 0.1% sodium azide as a preservative in 0.1M PBS until sinking to the bottom of the container, indicating they had been adequately saturated. The brains were cut into 30 µm thick coronal sections and stored at 4 °C in PBS with 0.1% sodium azide until use.

### Immunofluorescence

**Fig. 1:**
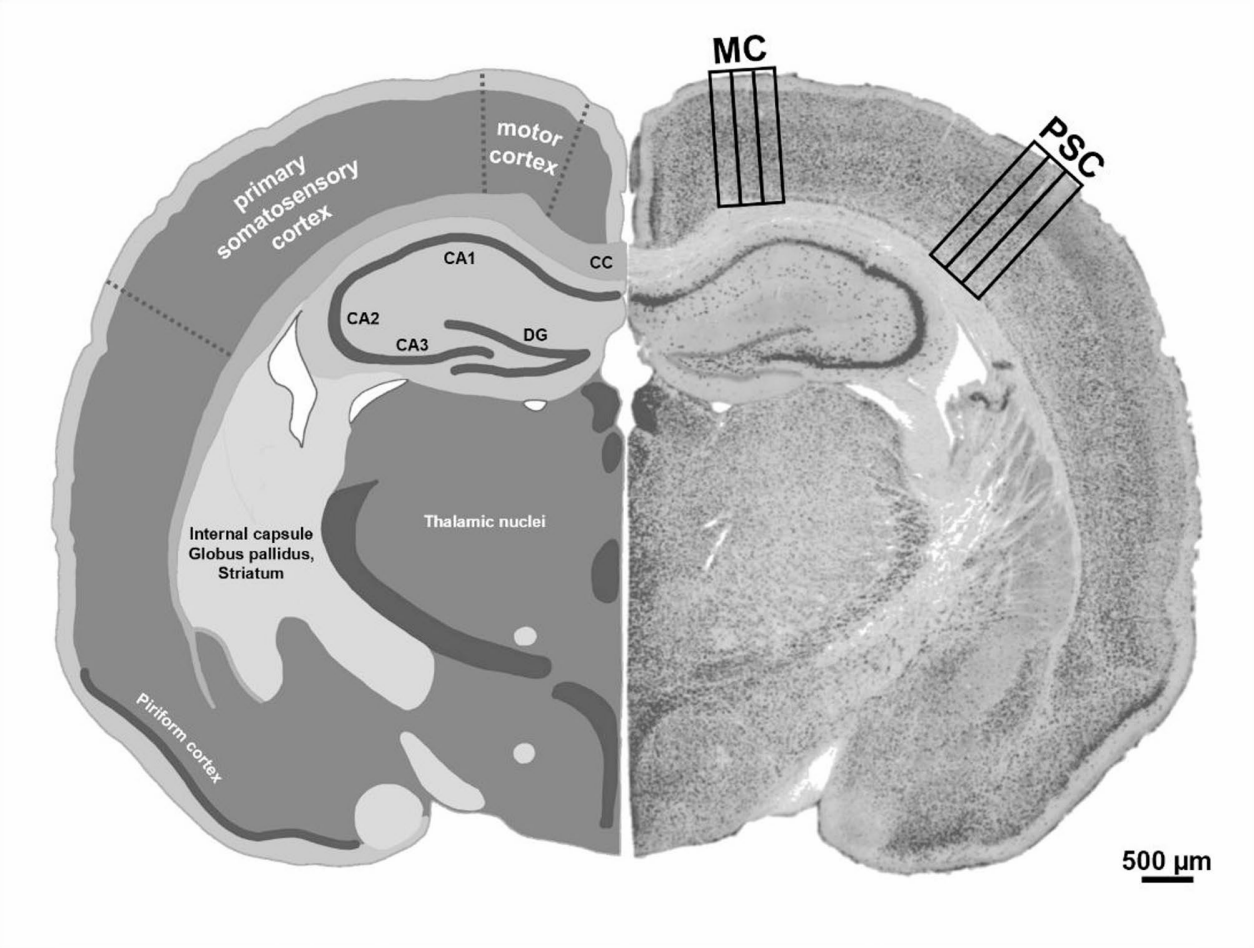
Overview of the coronary cross-section of a Syrian hamster brain, shown schematically on the left and stained with the neuron marker anti-HuC/D on the right. Marked areas indicate the regions used for examination of the motor cortex (MC) and primary somatosensory cortex (PSC). Each area was subdivided into three regions of interest (ROI), each measuring 200 µm in width and spanning all six neocortical layers. *A Stereotactic Atlas of The Golden Hamster Brain* by *Morin and Wood, 2001*^69^ was consulted for neuroanatomical orientation to determine regions of interest. Legend: CC = Corpus callosum, CA1/2/3 = Region 1/2/3 of the Cornu ammonis of the Hippocampus, DG = Dentate gyrus of the Hippocampus.

For immunofluorescence procedures, per animal, three consecutive free-floating brain sections were selected at the level shown in Fig. 1 with the help of A Stereotactic Atlas of The Golden Hamster Brain by Morin and Wood, 2001^69^. The slices were then bisected to allow for the application of two different triple stains to the three consecutive hemispheres. The hemispheres were rinsed twice (10 min per rinse) in Tris-buffered saline with 0.02% Tween (TBS-T) and incubated for 30 min in a solution of 2% hydrogen peroxide and 60% methanol. Sections were rinsed again in TBS-T and then incubated for 60 min in blocking solution of 2% normal donkey serum (Biozol Diagnostika Vertrieb GmbH, Eching, Germany) and 0.3% Triton X-100, diluted in TBS. Subsequently, they were incubated for 48 h at 4 °C in the same blocking solution containing one of the following two combinations of primary antibodies: one set of consecutive hemispheres was incubated with a polyclonal rabbit anti-aggrecan (1:500; Merck Millipore, Billerica, Massachusetts, USA; AB1031), mouse IgG1 anti-phospho-tau (1:900; Invitrogen AG, Carlsbad, California, USA; AT8), and mouse IgG2b anti-HuC/D (1:300; Invitrogen AG; 16A11) antibodies. The second set of hemispheres was incubated with the same rabbit anti-aggrecan and mouse IgG1 anti-phospho-tau antibodies but with an additional polyclonal guinea pig anti-parvalbumin antibody (1:1000; Synaptic Systems GmbH, Göttingen, Germany; 195 004) instead of the mouse IgG2b anti-HuC/D antibody. After rinsing in TBS-T three times, the sections were incubated for 60 min at room temperature in a solution of 2% bovine serum albumin (Serva Electrophoresis GmbH, Heidelberg, Germany) and 0.01% sodium azide, diluted in TSB-T, with different biotinylated secondary antibodies, depending on the combination. For the first combination, biotinylated donkey anti-mouse IgG2b antibody (1:800; Dianova GmbH, Hamburg, Germany) was applied, whereas biotinylated donkey anti-guinea pig (1:1000; Dianova GmbH) antibody was employed for the second combination. Subsequently, the sections were rinsed three times in TBS-T and incubated for 60 min at room temperature in the same blocking solution with Cy3-conjugated donkey anti-rabbit (1:1000; Dianova GmbH), Cy5-conjugated donkey anti-mouse IgG1 (1:800, Dianova GmbH) secondary antibodies, and Cy7-conjugated streptavidin (1:500; Dianova GmbH). After rinsing in TBS-T twice more, the nuclear stain Hoechst 33342 (1:5000; Invitrogen AG), diluted in TSB, was applied for 30 min. Finally, sections were rinsed in TBS twice, mounted on gelatin-coated slides, dehydrated in ascending alcohol series (70, 85, 95 and 100%), cleared in toluene and coverslipped with Entellan® (Merck Millipore).

### Image acquisition and processing

Using a Zeiss Axio Scan Z.1 slide scanner, fluorescence microscopic image stacks were recorded at 0.42 µm intervals over a span of 45 µm. The stacks were optimized for image segmentation by applying the „"extended depth of focus" function in the "variance"” mode to create single composite images. Subsequently, areas in the motor cortex and primary somatosensory cortex were selected according to the aforementioned atlas^69^. Each area was then subdivided into three regions of interest (ROI), measuring 200 µm in width and spanning all six neocortical layers (Fig. 1).

Images in Fig. 5 were generated using a confocal laser scanning microscope, the Zeiss LSM 880 NLO with fast Airyscan, to enhance the visibility of hyperphosphorylated tau protein within perineuronal nets for purposes of presentation only. All quantitative analyses were conducted on image stacks acquired via the Axio Scan slide scanner, as previously described.

### Image segmentation

**Fig. 2:**
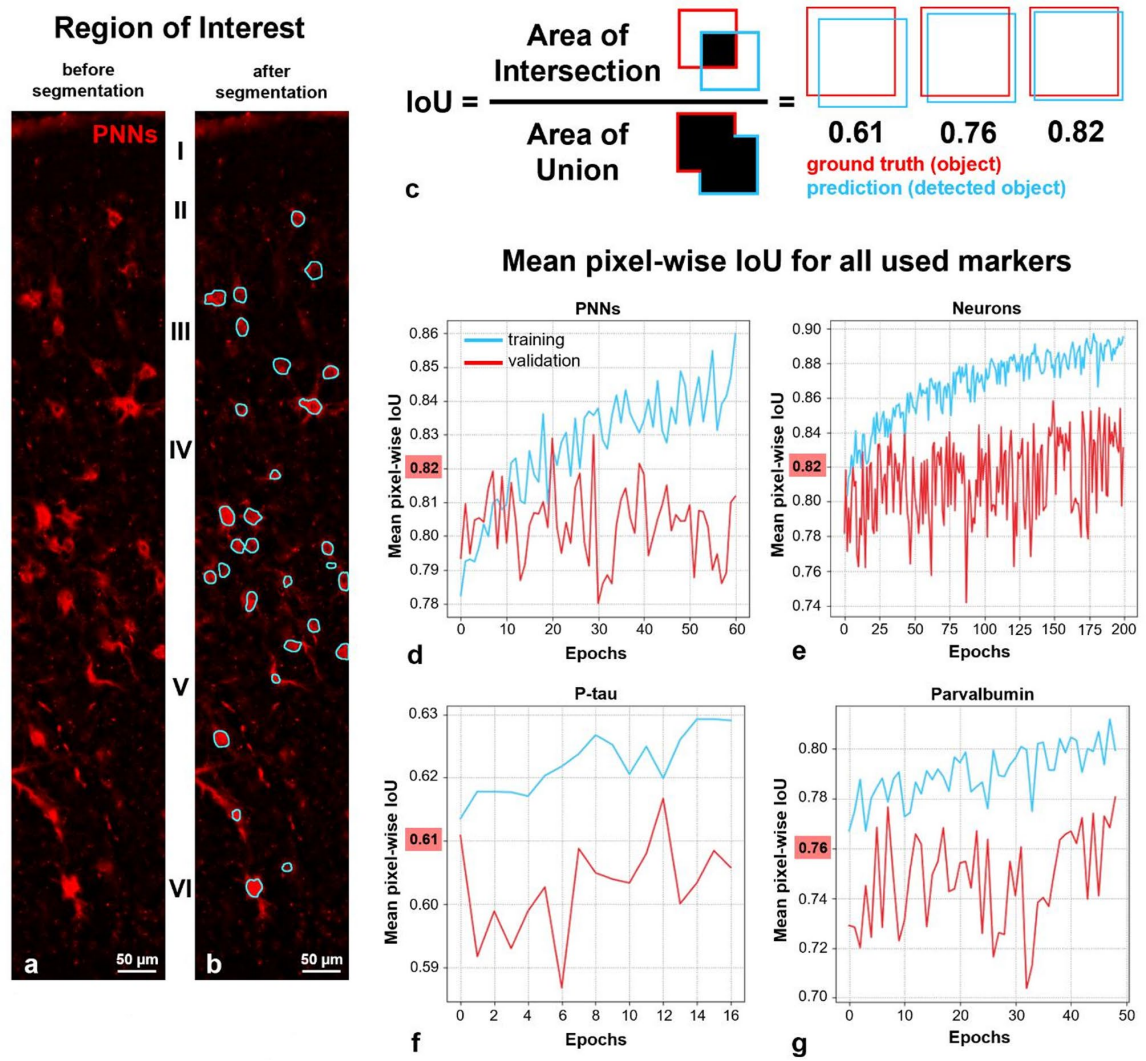
Visual and quantitative representation of segmentation results and their evaluation. (**a)** and (**b**) Comparison of the region of interest (ROI) before (**a**) and after (**b**) segmentation using an arivis Cloud-trained artificial intelligence model for the detection of perineuronal nets (PNNs). Each ROI has a width of 200 µm and spans all six neocortical layers (I-VI). (**c**) Schematic illustration of the principle of intersection over union (IoU) as the ratio of the area of overlap to the area of union of a ground truth (red box) and a prediction (blue box). The IoU is a measure that demonstrates the degree of alignment between a model’s prediction and the ground truth, with values ranging from 0 to 1. A value approaching 1 indicates a greater degree of similarity between the model’s prediction and the ground truth. Three results corresponding to the IoU values relevant to this research are represented: 0.61 for phosphorylated tau protein (p-tau), 0.76 for parvalbumin, and 0.82 for PNNs and neurons. Values greater than 0.5 are typically deemed acceptable, while values greater than 0.8 are regarded as excellent. (**d**)–(**g**) Mean pixel-wise IoU as a function of epochs for each staining used to train a model: PNNs (**d**), neurons (**e**), p-tau (**f**), and parvalbumin (**g**). In the context of machine learning, epochs represent the number of times a model passes through the entire training dataset. IoU values visualized in c are highlighted in red on the y-axis. The models presented were selected for the final segmentation based on the following criteria: (1) the plateauing (**d**) or only marginal further improvement (**e**)–(**g**) of the validation curve, (2) the incipient divergence of the training and validation curves, which can indicate the onset of overfitting, and (3) subsequent visual inspection (**a** and **b**). Graphs were provided by arivis Cloud. Legend: PNNs = perineuronal nets, IoU = Intersection over Union, p-tau = hyperphosphorylated tau protein.

For image segmentation, the online Zeiss application arivis Cloud, formerly Apeer^70^, was employed to generate artificial intelligence models capable of detecting the required structures with greatest possible reliability. The web service allowed for the training of deep learning models, which were subsequently used for the streamlined segmentation of images in the Zen Blue application Intellesis by Zeiss. This approach enabled a more time- and cost-efficient image analysis than would have been possible with manual counting of this extent. A representative illustration of a region of interest (ROI) before and after segmentation by an arivis Cloud-trained model for recognizing perineuronal nets is provided in Fig. 2a and b. Each ROI has a width of 200 µm and spans all six layers of the neocortex.

To ensure the high quality of the analysis, it was necessary to assess the precision and reliability of the respective models. The evaluation was conducted using the parameters loss, accuracy, and intersection over union (IoU, Fig. 2c–g) provided by arivis Cloud, in addition to the visual inspection (Fig. 2a and b). The IoU is a metric that quantifies the degree of alignment between a model’s prediction and the ground truth. This enables the assessment of a model’s object recognition performance, expressed as a value between 0 and 1. A value closer to 1 indicates a greater degree of agreement between the model’s prediction and the ground truth. Therefore, the closer the IoU is to 1, the better the model recognizes what it is supposed to recognize. The term “ground truth” refers to the target structure that has been identified and labelled as such by the annotator, e.g. a perineuronal net. In general, values greater than 0.5 are regarded as acceptable^71^, while those exceeding 0.8 are deemed to be excellent. The IoU is a measure of the quality of a model’s recognition of individual objects^72^, whereas accuracy refers to the proportion of correct predictions in all predictions. Conversely, the loss function indicates the deviation of the model’s predictions from the ground truth and its objective is to be minimized during the training process. When all quality parameters were considered collectively, models with IoU values of 0.61 (hyperphosphorylated tau protein), 0.76 (parvalbumin), and 0.82 (perineuronal nets and neurons) were ultimately selected for use. Those values are illustrated schematically in Fig. 2c, and as graphs of the actual models in Fig. 2d–g, provided by arivis Cloud. The plots visualize the evolution of the IoU over multiple epochs, whereby an epoch describes a complete pass of the model through the entire training data set. Before the actual training, the annotated data set is automatically split into a training data set and a validation data set. The model is then trained exclusively with data from the training data set, which constitutes approximately 80% of the entire annotated data set^73^. To assess the model’s ability to handle previously unseen data, it is evaluated using the validation data set and thus, the ground truth. This results in two curves for each model, one representing the performance of the model on the training data set and the other representing the performance on the validation data set. The performance on the validation data set demonstrates the extent to which the model can be applied to previously unknown data. If the model performs significantly better on the training data than on the validation data and/or if the performance of the model declines after an initial improvement, there is a risk of overfitting to the training data set. Overfitting should be avoided as it reduces the generalizability of the model^74^.

To minimize the occurrence of overfitting in the present work, the version of the model was selected as the final version at which the curves for the IoU, accuracy, and loss had just begun to plateau: the graphs presented here demonstrate no (Fig. 2d) or only minimal further increases (Fig. 2e–g) in the training and validation curves over several epochs. Furthermore, the training curve begins to diverge from the validation curve, possibly indicating incipient overfitting. Given the marginal differences between the two curves and the model’s performance on the validation data set, which is nearly on par with its performance on the training data set, the models presented here were utilized for the final segmentation. In order to minimize the potential for misidentification of large net-ensheathed dendrites as false positives, only PNNs with a diameter greater than 5 µm were counted.

### Statistical analysis

All statistical analyses were performed using GraphPad Prism version 10.3.1 for Windows.

To avoid pseudoreplication, the data of each region of interest (ROI) were first aggregated by calculating the mean value of all data points associated with a single animal and/or region within that animal. The number of ROIs per animal and region varied, depending on the specific staining, with either 9 or 18 ROIs per animal and region (i.e., technical replicates). As there were three animals per group (i.e., biological replicates), this procedure resulted in three means for each group or region, which were subsequently analyzed statistically. No data points were excluded from the analysis.

Given that, due to low power when performed on small sample sizes, even stark deviations from a normal distribution often remain undetected by normality tests^75,76^, it is not recommended to test for normality on small sample sizes for the purpose of selecting between nonparametric tests and those that assume normality, such as Student’s and Welch’s t-tests.

Therefore, we assumed a normal distribution in our data due to previous research and experience in our field and laboratory and, additionally, inspected q-q plots visually to gauge possible distributional deviations from normality, as recommended by several authors^76,77^. Given that Welch’s t-test—also known as the t-test for unequal variances—proved to be very robust to deviations from normal distribution even in small sample sizes^75^, it was selected for the comparison of means between the euthermic and torpid groups. Furthermore, Welch’s t-test loses (almost) no power compared to Student’s t-test when variances are equal but outperforms Student’s t-test in controlling for type I and type II errors when variances are unequal^78,79^. As unequal variances cannot be ruled out with certainty, and given that testing for them is not advised for selecting between Student’s t-test and Welch’s t-test, as this can lead to inflated type I errors^80^, Welch’s t-test was the test of choice for the present work for comparison of means between groups.

For comparison of means between regions within the same group (e.g. the relative number of perineuronal nets in the motor cortex of euthermic animals compared to their primary somatosensory cortex), the paired t-test was employed. As the respective regions are necessarily paired by animal, the paired t-test was selected to more accurately account for the lack of independence.

The resulting data are given as mean ± standard deviation and displayed by means of SuperPlots as proposed by Lord et al. to visualize both biological (*N*) and technical replicates (*n*)^81^.

## Results

### PNN expression remains constant during torpor

**Fig. 3:**
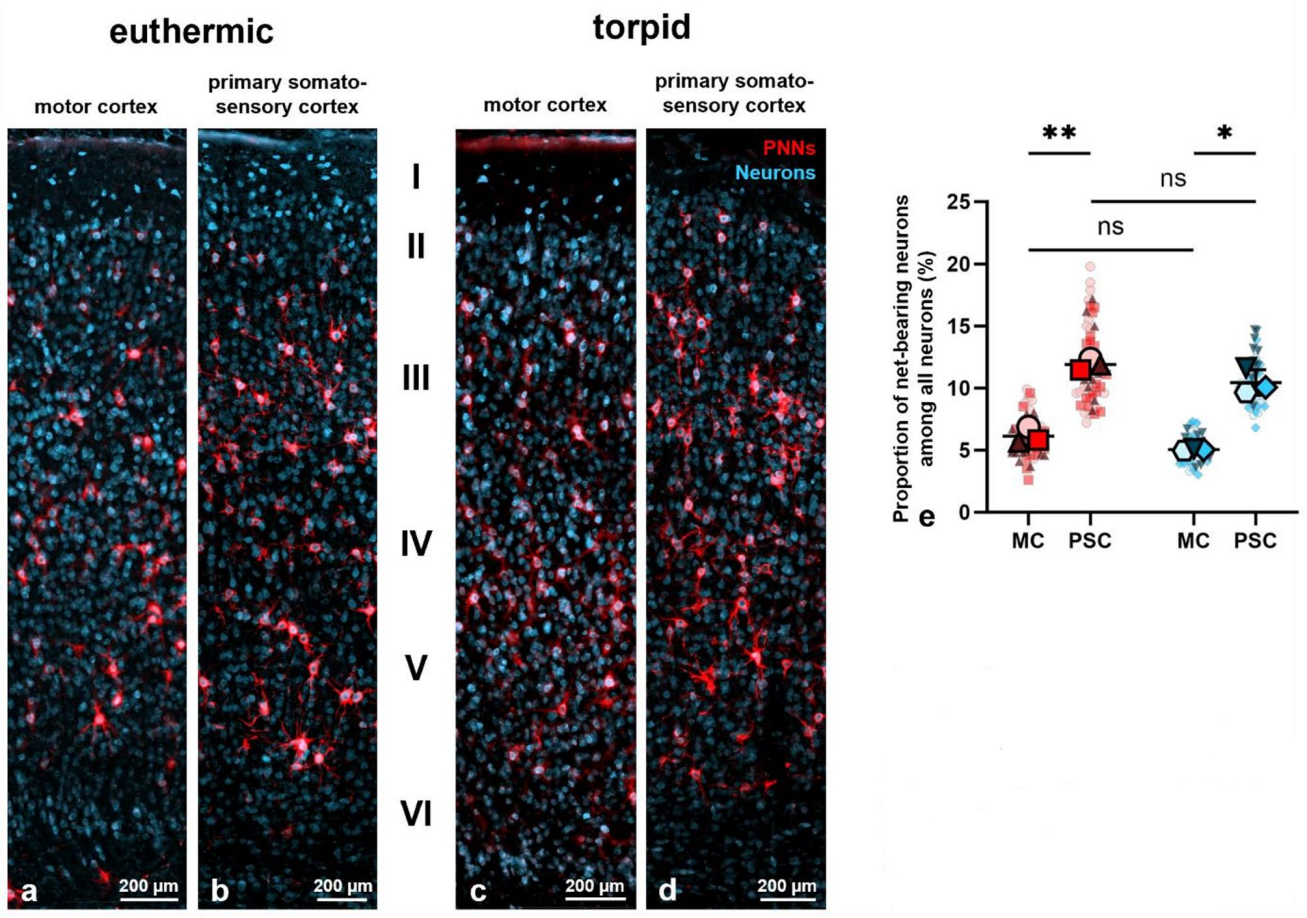
Distribution and proportion of net-bearing neurons among all neurons in the motor cortex (MC) and primary somatosensory cortex (PSC) of Syrian hamsters in both euthermic and torpid states. (**a**)–(**d**) Distribution of neurons (blue) and perineuronal nets (PNNs, red) in the motor cortex (**a**, **c**) and primary somatosensory cortex (**b**, **d**) of Syrian hamsters in the euthermic (**a**, **b**) and torpid (**c**, **d**) states across all six neocortical layers (I-VI). Although no discernible difference in PNN expression is evident between the two experimental conditions, the presence of PNNs appears to be more pronounced in the primary somatosensory cortex than in the motor cortex in both the euthermic and torpid states. (**e**) Quantification of the percentage (%) of net-bearing neurons in relation to all neurons in the motor cortex and primary somatosensory cortex in euthermic and torpid animals. The proportion of net-bearing neurons among all neurons was significantly higher in the primary somatosensory cortex than in the motor cortex, both in the euthermic state (*p* = *0.0016, N* = *3, n* = *27, paired t-test*) and during torpor (*p* = *0.0106, N* = *3, n* = *27, paired t-test*). No statistically significant difference was observed in the relative number of PNNs among all neurons between the euthermic and torpid states, in both the motor cortex (*p* = *0.1054, N* = *3, n* = *27, Welch’s t-test*) and the primary somatosensory cortex (*p* = *0.1216, N* = *3, n* = *27, Welch’s t-test*). Legend: PNNs = perineuronal nets, MC = motor cortex, PSC = primary somatosensory cortex, cener line = mean, error bars = standard deviation (SD), red = euthermic values, blue = torpid values, large symbols = biological replicates (N), small symbols = technical replicates (n), *p < 0.05, **p < 0.01, ***p < 0.001, ns = not significant.

Accumulation of hyperphosphorylated tau protein (p-tau) is a major pathological hallmark of certain neurodegenerative diseases known as tauopathies, such as Alzheimer’s disease^38–43^. In light of prior research indicating that neurons enwrapped by perineuronal nets (PNNs) rarely exhibit aggregates of p-tau^33–36^ and that net-bearing neurons are better protected from oxidative stress^26–29^, PNNs have been proposed to serve neuroprotective functions. However, given that p-tau is also present under non-pathological conditions, including during hibernation^66^, the phosphorylation of tau cannot be deemed pathological in and of itself. This leads to the central question of the present study, namely whether PNN-ensheathed neurons are also devoid of physiologically hyperphosphorylated tau protein, specifically during the torpor phase of the Syrian hamster. Therefore, we first identified any general differences in the distribution and quantity of PNNs between euthermic and torpid animals. Given the pronounced PNN expression observed in the neocortex and the documented silencing of this brain region during hibernation^64,65^, we analyzed the proportion of net-bearing neurons in relation to all neurons within the neocortex in both states (Fig. 3). This included neurons from both the motor cortex and the primary somatosensory cortex. Consequently, neurons were immunohistochemically stained with an anti-HuC/D antibody, while PNNs were detected using the anti-aggrecan antibody AB1031, as aggrecan is the predominant lectican found in mature cortical PNNs^4,5^. Representative images of PNNs ensheathing neurons in the examined brain regions are provided in Fig. 3a–d.

As there appeared to be a greater abundance of PNNs in the primary somatosensory cortex (Fig. 3b and d) than in the motor cortex (Fig. 3a and c) in both states, we conducted a quantitative analysis to verify this visual discrepancy (Fig. 3e). Indeed, the data demonstrate that the proportion of net-bearing neurons among all neurons was significantly higher in the primary somatosensory cortex than in the motor cortex, both in the euthermic state (*motor cortex: 6.1* ± *0.7%; primary somatosensory cortex: 11.9* ± *0.4%, p* = *0.0016 (N* = *3, n* = *54, paired t-test)*) and during torpor (*motor cortex: 5.0* ± *0.1%; primary somatosensory cortex: 10.5* ± *1.0%, p* = *0.0106 (N* = *3, n* = *54, paired t-test)*). On average, approximately 6% (*5.6* ± *0.7% (N* = *6, n* = *108)*) of neurons exhibited PNNs in the motor cortex and 11% (*11.2* ± *1.1% (N* = *6, n* = *108)*) in the primary somatosensory cortex, irrespective of the experimental condition. Taken together, approximately 8% (*8.4* ± *0.8% (N* = *6, n* = *216)*) of neurons in the examined neocortical regions were ensheathed by PNNs, and the prevalence of net-bearing neurons was about 50% (*50.1* ± *4.0% (N* = *6, n* = *108)*) higher in the primary somatosensory cortex than in the motor cortex (*euthermic: 48.6* ± *4.0% (N* = *3, n* = *54); torpid: 51.5* ± *4.1% (N* = *3, n* = *54)*). Given this regional discrepancy in PNN expression, subsequent investigations were conducted separately for the motor cortex and the primary somatosensory cortex to identify region-specific variations.

Statistical analysis further demonstrated no significant difference in the proportion of PNNs among neurons observed in euthermic versus torpid animals in the motor cortex (*euthermic: 6.1* ± *0.7%, torpid: 5.0* ± *0.1%, p* = *0.1054 (N* = *3, n* = *54, Welch’s t-test)*) and the primary somatosensory cortex (Fig. 3e; *euthermic: 11.9* ± *0.4%, torpid: 10.5* ± *1.0%, p* = *0.1216 (N* = *3, n* = *54, Welch’s t-test)*).

In conclusion, while the number of PNNs was higher in the primary somatosensory cortex than in the motor cortex in both experimental conditions, it remained constant during hibernation compared to euthermic conditions.

### Net-bearing neurons are more likely to exhibit hyperphosphorylated tau protein during torpor than the overall neuronal population

**Fig. 4:**
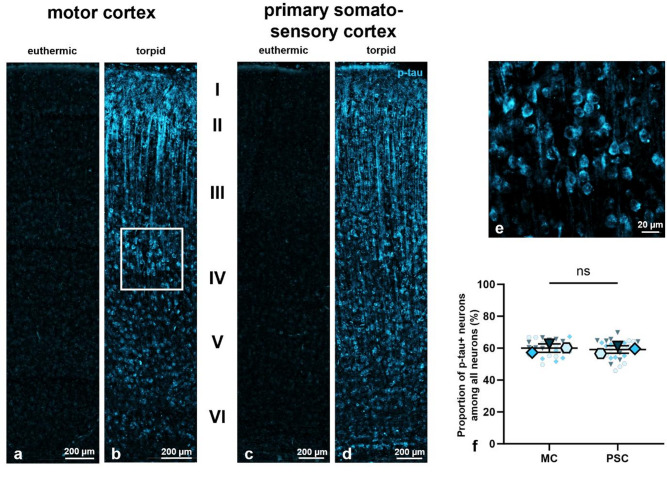
Distribution and proportion of neurons expressing hyperphosphorylated tau protein (p-tau) in the motor cortex (MC) and primary somatosensory cortex (PSC) of both euthermic and torpid Syrian hamsters. (**a**)–(**d**) Distribution of p-tau+ neurons (blue) in the motor cortex (**a**, **b**) and primary somatosensory cortex (**c**, **d**) of Syrian hamsters in the euthermic (**a**, **c**) and torpid (**b**, **d**) states across all six neocortical layers (I-VI). P-tau is exclusively present during torpor, shown at a higher magnification in (**e**). (**e**) Squared zone in b shown at a higher magnification. AT8-immunoreactivity is present in the somatodendritic compartment of neurons within the motor cortex of torpid animals. (**f**) Quantification of the percentage (%) of p-tau+ neurons in relation to all neurons in the motor cortex compared to the primary somatosensory cortex of torpid animals. No statistically significant regional difference was observed during torpor (*p* = *0.6610, N* = *3, n* = *27, paired t-test)*). Legend: p-tau = hyperphosphorylated tau protein, MC = motor cortex, PSC = primary somatosensory cortex, center line = mean, error bars = standard deviation (SD), blue = tropid values, large symbols = biological replicates (N), small symbols = technical replicates (n), *p < 0.05, **p < 0.01, ***p < 0.001, ns = not significant.

Neurons enwrapped by perineuronal nets (PNNs) have been documented to be devoid of hyperphosphorylated tau protein (p-tau) in the brains of Alzheimer’s disease patients^34–36^. Furthermore, p-tau has been previously reported to occur during torpor in several species, including the Syrian hamster^66^. To investigate the relationship between the expression of PNNs and the prevalence of p-tau under physiological conditions, we initially confirmed the presence of p-tau in our hamster brain samples. For this purpose, we employed the anti-p-tau antibody AT8, which specifically recognizes tau phosphorylated at the phospho-sites Ser202 and Thr205^82^. However, given that phosphorylation has been demonstrated at numerous other sites^59,60,66^, we assumed that AT8 immunoreactivity could serve as a surrogate marker for general *hyper*phosphorylation in our case. Immunohistochemical staining of neocortical sections from both euthermic and torpid animals clearly showed that p-tau was present exclusively during torpor (Fig. 4a–d). In order to determine the number of neurons that expressed p-tau during hibernation, a neuron was classified as p-tau positive (p-tau+) when p-tau was present in its soma. This classification was applied irrespectively of whether the neuron also or solely exhibited axonal p-tau, as p-tau expression was most consistent in the somatodendritic compartment (Fig. 4e). We then estimated the proportion of p-tau+ neurons in torpid animals, focusing on regional differences between the motor cortex and primary somatosensory cortex. However, statistical analysis revealed no significant difference in the proportion of p-tau+ neurons among all neurons in the motor cortex (*60.1* ± *2.6% (N* = *3, n* = *27)*) and primary somatosensory cortex (*59.2* ± *2.4%, p* = *0.6610 (N* = *3, n* = *27, paired t-test)*) during torpor (Fig. 4f). Overall, the data indicate that a majority of approximately 60% (*59.6* ± *2.0% (N* = *3, n* = *54)*) of neocortical neurons in torpid Syrian hamsters displayed somatodendritic hyperphosphorylated tau protein.

**Fig. 5:**
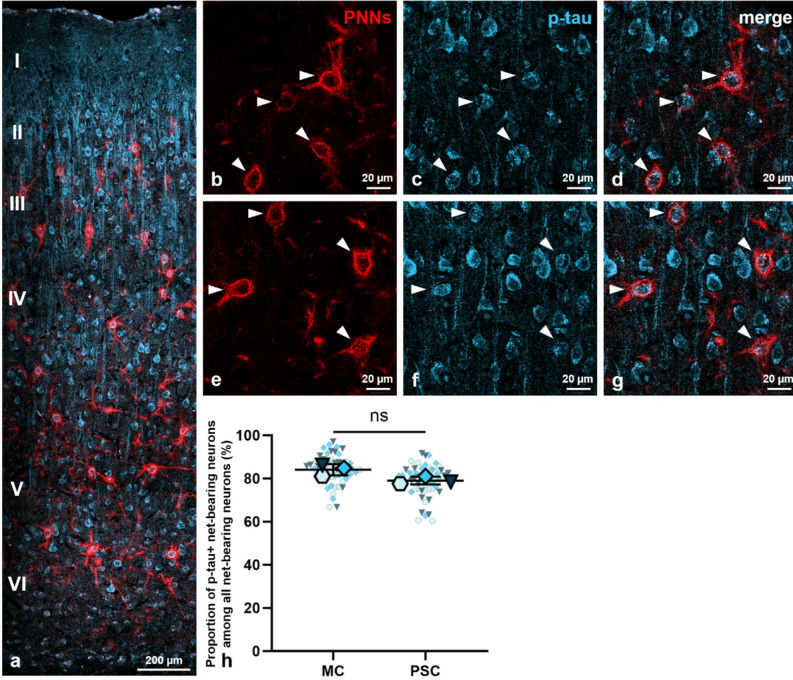
Net-bearing neurons display hyper-phosphorylated tau protein (p-tau) during torpor. (**a**) Distribution of perineuronal nets (PNNs, red) enwrapping p-tau+ neurons (blue) in the motor cortex across all six neocortical layers (I-VI) during torpor. (**b**)–(**g**) Representative images of p-tau inside net-bearing neurons (arrow heads) at a higher magnification. (**h**) Quantification of the percentage (%) of p-tau+ net-bearing neurons in relation to all net-bearing neurons in the motor cortex compared to the primary somatosensory cortex of torpid animals. No statistically significant regional difference was observed during torpor (*p* = *0.0672, N* = *3, n* = *54, paired t-test*). Legend: PNNs = perineuronal nets, p-tau = hyperphosphorylated tau protein, center line = mean, error bars = standard deviation (SD), red = euthermic values, blue = torpid values, large symbols = biological replicates (N), small symbols = technical replicates (n), *p < 0.05, **p < 0.01, ***p < 0.001, ns = not significant.

Given the documented absence of p-tau in neurons expressing PNNs in individuals with Alzheimer’s disease^34–36^, we next focused specifically on the population of net-bearing neurons. To investigate whether these neurons are devoid of p-tau during hibernation, we employed and analyzed a double-staining of both PNN (anti-aggrecan antibody AB1031) and p-tau (anti-p-tau antibody AT8) markers within the neocortex. As p-tau was not observed in euthermic animals, as previously shown in Fig. 4a and c, this analysis was conducted exclusively on torpid animals. To our surprise, immunofluorescent observations clearly demonstrated that PNN-ensheathed neurons were not free of p-tau during torpor, as illustrated in representative images in Fig. 5a–g. Indeed, statistical analysis revealed that approximately 82% (*81.6* ± *1.9% (N* = *3, n* = *54)*) of net-bearing neurons exhibited p-tau during torpor, without significant regional differences between the motor cortex (*84.1* ± *2.7% (N* = *3, n* = *54)*) and primary somatosensory cortex (*79.1* ± *1.8%, p* = *0.0672 (N* = *3, n* = *54, paired t-test)*) of torpid animals (Fig. 5h).

In summary, our data demonstrate that approximately 8% of all neocortical neurons during hibernation were covered by PNNs. While roughly 82% of those net-bearing cells displayed hyperphosphorylated tau protein during torpor, only about 60% of all neocortical neurons exhibited p-tau. These results thus led to the unexpected finding that the prevalence of p-tau was higher among PNN-enwrapped neurons than among the overall neuronal population within the neocortex of torpid animals. Consequently, the subset of net-bearing neurons was more likely to express p-tau than the neocortical neurons in their entirety. Given this surprising outcome, we next aimed to further characterize the subset of net-bearing neurons regarding their association with p-tau formation.

### Hyperphosphorylated tau protein is strongly linked to parvalbumin expressing net-bearing neurons

The aim of the following step was to identify a feature of net-bearing cells that could explain the high prevalence of hyperphosphorylated tau protein (p-tau) among these neurons. We thus analyzed the association between perineuronal nets (PNNs) and fast-firing parvalbumin (PV) expressing inhibitory interneurons for the following two reasons: (i) The majority of neurons and net-bearing neurons displayed p-tau during hibernation in the present study, suggesting that tau hyperphosphorylation may be explicitly required in certain cell types during torpor. P-tau has been reported to reduce neuronal activity and excitability in tauopathy mouse models^83–85^, and hibernation with the concomitant cessation of cortical activity serves to minimize energy expenditure. Therefore, fast-firing, highly active neurons may represent a potential target for the suppression of excitability during torpor. The largest subset of inhibitory interneurons demonstrating such fast-spiking behavior comprises approximately 40% of all inhibitory interneurons and is reliably marked by PV^86–88^. (ii) In numerous species, a close correlation has been observed between net-bearing neurons and PV+ interneurons^14,89^. Therefore, PV expression may be a shared feature of p-tau+ and net-bearing neurons during torpor, identifying a particular subset of PNN-ensheathed cells that is especially likely to exhibit p-tau. However, to our knowledge, no data has been made available thus far to confirm the interrelation of PV+ interneurons and PNNs in the neocortex of the Syrian hamster, and evidence on hibernation-dependent changes in PV-expression is scarce and inconclusive^90,91^.

**Fig. 6:**
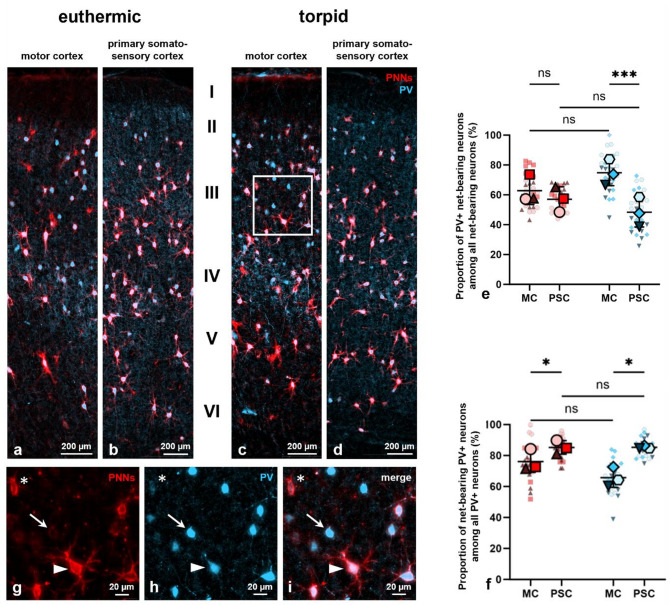
Distribution and proportion of net-bearing neurons and parvalbumin+ (PV+) interneurons in the motor cortex (MC) and primary somatosensory cortex (PSC) of Syrian hamsters in both euthermic and torpid states. (**a**)–(**d**) Distribution of perineuronal nets (PNNs, red) and PV+ interneurons (blue) in the motor cortex (**a**, **c**) and primary somatosensory cortex (**b**, **d**) of Syrian hamsters in the euthermic (**a**, **b**) and torpid (**c**, **d**) states across all six neocortical layers (I-VI). (**e**) Quantification of the percentage (%) of PV+ interneurons among net-bearing neurons in the motor cortex and primary somatosensory cortex in euthermic and torpid animals. No statistically significant difference was observed in the proportion of PV+ interneurons among net-bearing neurons between the euthermic and torpid states, in both the motor cortex (*p* = *0.1788, N* = *3, n* = *27, Welch’s t-test*) and the primary somatosensory cortex (*p* = *0.3113, N* = *3, n* = *27, Welch’s t-test*). However, the number of PV+ interneurons among net-bearing neurons was significantly higher in the motor cortex than in the primary somatosensory cortex in torpid animals (*p* = *0.0009, N* = *3, n* = *27, paired t-test*). While a similar trend was evident in euthermic animals, no statistically significant difference was observed (*p* = *0.5000, N* = *3, n* = *27, paired t-test*). (**f**) Quantification of the % of PNN-ensheathed neurons among PV+ interneurons in the motor cortex and primary somatosensory cortex in euthermic and torpid animals. No statistically significant difference was observed in the proportion of net-bearing PV+ interneurons among all PV+ interneurons between the euthermic and torpid states, in both the motor cortex (*p* = *0.1295, N* = *3, n* = *27, Welch’s t-test*) and the primary somatosensory cortex (*p* = *0.9916, N* = *3, n* = *27, Welch’s t-test*). The number of net-bearing PV+ interneurons among all PV+ interneurons was significantly higher in the primary somatosensory cortex than in the motor cortex, both in the euthermic state (*p* = *0.0411, N* = *3, n* = *27, paired t-test*) and during torpor (*p* = *0.0243, N* = *3, n* = *27, paired t-test*). (**g**)–(**i**) Squared zone in (**c**) shown at a higher magnification. Net-bearing neurons containing (arrow heads) and devoid of PV (asterisks)as well as PV+ interneurons ensheathed by (arrow heads) and free of PNNs (arrows), are visible. Legend: PNNs = perineuronal nets, PV= parvalbumin, MC = motor cortex, PSC = primary somatosensory cortex, center line = mean, error bars = standard deviation (SD), red = euthermic values, blue = torpid values, large symbols = biological replicates (N), small symbols = technical replicates (n), *p < 0.05, **p < 0.01, ***p < 0.001, ns = not significant.

Thus, to investigate the potential link between PNNs, PV, and p-tau, we initially characterized possible changes in PV expression among net-bearing neurons as well as the expression of PNNs by PV+ interneurons during hibernation. We therefore conducted an immunohistochemical double-staining using the polyclonal anti-parvalbumin antibody 195 004 to label PV+ interneurons and the anti-aggrecan antibody AB1031 to detect aggrecan-expressing PNNs (Fig. 6a–d, g–i). The resulting data indicate that there was no statistically significant difference between euthermic and torpid animals regarding the proportion of PV+ interneurons among net-bearing neurons in both neocortical regions (Fig. 6e): while in the motor cortex, approximately 63% of net-bearing neurons expressed PV in euthermic (*62.8* ± *9.4% (N* = *3, n* = *27)*) and 75% in torpid animals (*74.8* ± *8.6% (N* = *3, n* = *27*), this deviation did not reach statistical significance (*p* = *0.1788, N* = *3, n* = *27, Welch’s t-test)*). A similar pattern was observed in the primary somatosensory cortex, where approximately 57% of net-bearing neurons exhibited PV in euthermic (*57.1* ± *8.4% (N* = *3, n* = *27)*) and 48% in torpid animals (*48.3* ± *9.9%, p* = *0.3113 (N* = *3, n* = *27, Welch’s t-test)*). Furthermore, the number of PV+ interneurons ensheathed by PNNs compared to the total number of PV+ interneurons did also not differ between euthermic and torpid animals in either the motor cortex (*euthermic: 76.2* ± *7.0% (N* = *3, n* = *27); torpid: 65.7* ± *6.3%, p* = *0.1295 (N* = *3, n* = *27, Welch’s t-test)*) or the primary somatosensory cortex (Fig. 6f*; euthermic: 85.2* ± *4.4% (N* = *3, n* = *27); torpid: 85.3* ± *1.0%, p* = *0.9916 (N* = *3, n* = *27, Welch’s t-test)*).

However, the data demonstrate regional differences between the motor cortex and primary somatosensory cortex regarding the proportion of PV+ net-bearing neurons (Fig. 6e) as well as the number of PV+ interneurons exhibiting PNNs (Fig. 6f). Accordingly, the number of PV+ interneurons among net-bearing neurons was significantly higher in the motor cortex (*74.8* ± *8.6% (N* = *3, n* = *27)*) compared to the primary somatosensory cortex (*48.3* ± *9.9%, p* = *0.0009 (N* = *3, n* = *27, paired t-test)*) in torpid animals (Fig. 6e). Although no statistically significant difference was observed in euthermic animals, a similar trend was evident (*motor cortex: 62.8* ± *9.4% (N* = *3, n* = *27); primary somatosensory cortex: 57.1* ± *8.4%, p* = *0.5000 (N* = *3, n* = *27, paired t-test)*).

Furthermore, the proportion of PV+ interneurons enwrapped by PNNs among all PV+ interneurons was found to be significantly higher in the primary somatosensory cortex than in the motor cortex, both in the euthermic state (*motor cortex: 76.2* ± *7.0%; primary somatosensory cortex: 85.2* ± *4.4%, p* = *0.0411 (N* = *3, n* = *27, paired t-test)*) and during torpor (Fig. 6f*; motor cortex: 65.7* ± *6.3%; primary somatosensory cortex: 85.3* ± *1.0%, p* = *0.0243 (N* = *3, n* = *27, paired t-test)*). These findings align with those presented in Fig. 3, which indicate that a greater proportion of PNN-ensheathed neurons were present in the primary somatosensory cortex than in the motor cortex.

In conclusion, regional differences in PV expression associated with PNNs were observed in the motor cortex compared to the primary somatosensory cortex in both the euthermic and torpid states. While the expression of PV among net-bearing neurons was significantly higher in the motor cortex than in the primary somatosensory cortex in torpid animals only, the number of PNNs among PV+ interneurons was found to be elevated in the primary somatosensory cortex compared to the motor cortex in both states. Given that these regional discrepancies were largely comparable between euthermic and torpid animals and that no significant difference was observed between the two states, we conclude that PNN-associated PV expression remained constant throughout hibernation. In both experimental conditions, the majority of net-bearing neurons were found to express PV, while the majority of PV+ interneurons were also observed to exhibit PNNs. It is, however, noteworthy that not all PV+ interneurons exhibited aggrecan-expressing PNNs, and not all net-bearing neurons displayed PV (Fig. 6g–i), which is consistent with findings observed in other species^89,92^. In conclusion, the data demonstrate a strong correlation between net-bearing neurons and PV+ inhibitory interneurons in the neocortex of the Syrian hamster in both the euthermic and torpid states, thereby corroborating findings in other species.

The high prevalence of PV and p-tau among net-bearing neurons renders the subpopulation of PV+ net-bearing neurons a potential subset of net-bearing neurons with an exceptionally high prevalence of p-tau formation during torpor. Therefore, we proceeded to investigate whether p-tau was particularly abundant in PV+ net-bearing neurons and PV+ interneurons in general, with a view to determining whether PV could serve as an indicator of tau hyperphosphorylation during hibernation.

**Fig. 7:**
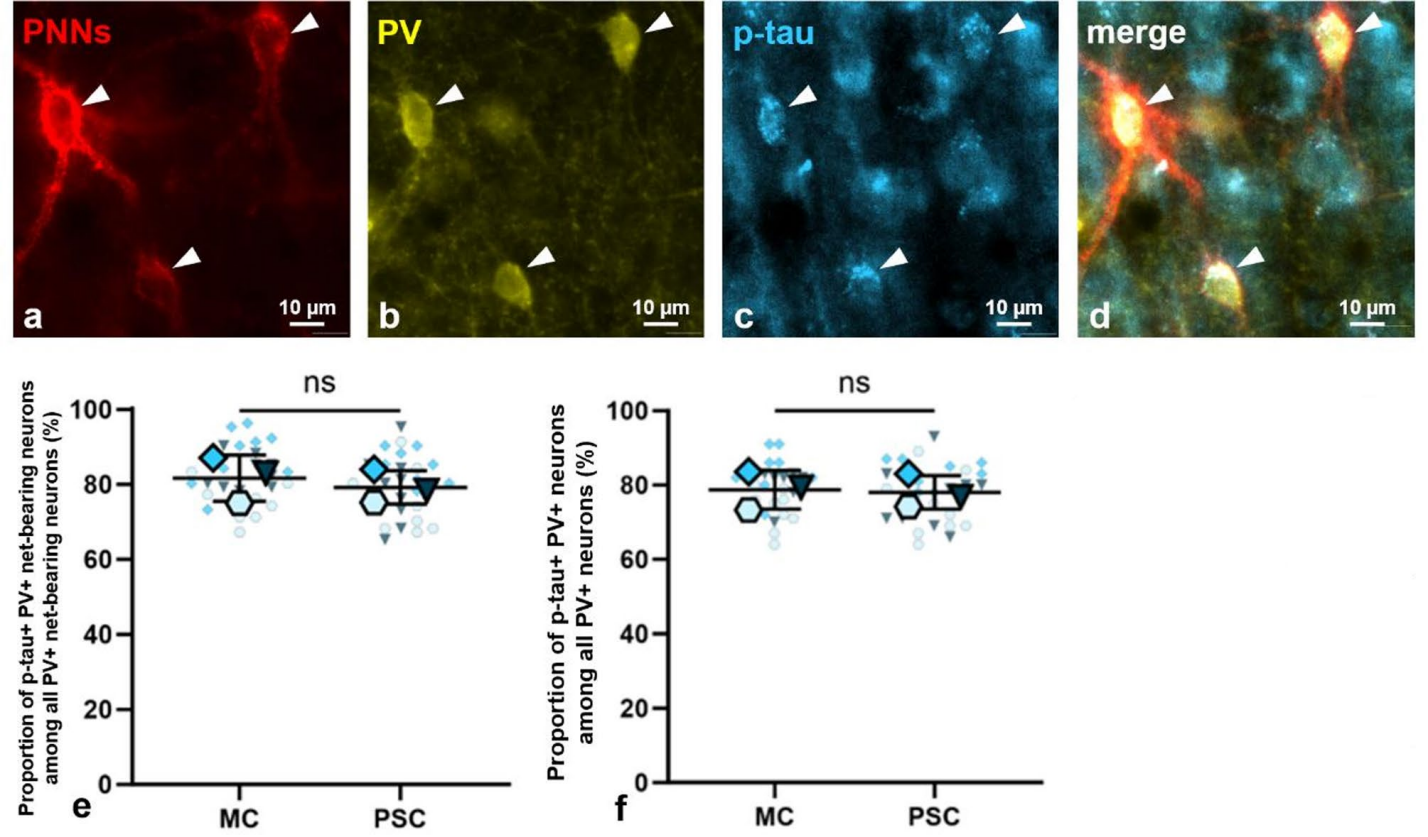
Hyperphosphorylated tau protein (p-tau) among parvalbumin (PV) expressing net-bearing neurons. (**a**)–(**d**) Representative images of net-bearing neurons (red) exhibiting PV (yellow) and p-tau (blue, arrow heads). (**e**) Quantification of the percentage (%) of p-tau+ PV+ net-bearing neurons among all PV+ net-bearing neurons in the motor cortex and primary somatosensory cortex of torpid animals. No statistically significant difference has been observed in the number of p-tau+ cells among PV+ net-bearing neurons between the motor cortex and primary somatosensory cortex during torpor (*p* = *0.2218, N* = *3, n* = *27, paired t-test*). (**f**) Quantification of the % of p-tau+ PV+ interneurons among all PV+ interneurons in the motor cortex and primary somatosensory cortex of torpid animals. No statistically significant difference was observed in the number of p-tau+ PV+ interneurons in relation to all PV+ interneurons, irrespective of whether they were ensheathed by a perineuronal net (PNN), between the motor cortex and the primary somatosensory cortex during torpor (*p* = *0.5622, N* = *3, n* = *27, paired t-test*). Legend: PNNs = perineuronal nets, PV = parvabulmin, p-tau = hyperphosphorylated tau protein, MC = motor cortex, PSC = primary somatosensory cortex, center line = mean, error bars = standard deviation (SD), blue = torpid values, large symbols = biological replicates (N), small symbols = technical replicates (n), *p < 0.05, **p < 0.01, ***p < 0.001, ns = not significant.

Statistical analysis of the corresponding triple-staining – using the anti-aggrecan antibody AB1031, the anti-p-tau antibody AT8 and the anti-parvalbumin antibody 195 004 to detect PNNs, p-tau and PV, respectively—revealed that, on average, approximately 81% (*80.5* ± *5.2% (N* = *3, n* = *54)*) of PV+ net-bearing neurons exhibited p-tau during torpor in both the motor cortex (*81.7* ± *6.2% (N* = *3, n* = *27)*) and the primary somatosensory cortex (*79.2* ± *4.5% (N* = *3, n* = *27)*), with no statistically significant regional difference (Fig. 7e; *p* = *0.2218, N* = *3, n* = *27, paired t-test*). Furthermore, no statistically significant difference was observed in the number of p-tau+ neurons among PV+ interneurons between the motor cortex (*78.8* ± *5.2% (N* = *3, n* = *27, paired t-test)*) and primary somatosensory cortex (*78.0* ± *4.5% (N* = *3, n* = *27, paired t-test)*) during torpor (Fig. 7f). Approximately 78% (*78.4* ± *4.8% (N* = *3, n* = *54)*) of PV+ interneurons displayed p-tau in both neocortical regions, irrespective of whether they were ensheathed by PNNs. Representative images of PV+ net-bearing neurons exhibiting p-tau are provided in Fig. 7a–d.

In conclusion, while about 60% of the overall neuronal population in the neocortex exhibited p-tau during the torpor phase of the Syrian hamster, a higher prevalence of p-tau was observed in net-bearing neurons (82%), PV+ net-bearing neurons (81%), and PV+ interneurons (78%), irrespective of their net-status. Therefore, the data clearly demonstrate a strong correlation between net-bearing neurons, PV, and p-tau, suggesting that the neuronal subsets of PV+ interneurons and (PV+) PNN-ensheathed neurons are especially likely to be affected by tau hyperphosphorylation during torpor. While the high prevalence of p-tau in the hibernating brain is not exclusively linked to the co-expression of PV and PNNs, the presence of either PNNs and/or PV does serve as an indicator for tau hyperphosphorylation in net-bearing neurons and fast-spiking PV+ interneurons.

## Discussion

The aim of this study was to investigate the correlation between the expression of perineuronal nets (PNNs) and the prevalence of hyperphosphorylated tau protein (p-tau) in the hibernating brain. During hibernation, hyperphosphorylation of tau protein occurs as a physiological and reversible process^58–60,66^, which is in contrast to tauopathies where hyperphosphorylation leads to the accumulation of pathological forms of tau protein^51,52^. In the brain of Alzheimer’s disease (AD) patients, net-bearing neurons were shown to be virtually devoid of any p-tau^33–36^, suggesting a neuroprotective function of PNNs. Here, we investigated by means of immunohistochemical analysis whether the formation of p-tau in net-bearing neurons is accordingly diminished during hibernation, a physiological condition associated with p-tau. Additionally, the link between PNNs, p-tau, and the calcium-binding protein parvalbumin (PV) was examined to potentially identify a shared feature of PNN-ensheathed and p-tau+ neurons.

There are three main findings in this study: (i) PNN expression, i.e. the number of PNNs among neurons, remained stable in the motor cortex (MC) and primary somatosensory cortex (PSC) during the torpor phase of the Syrian hamster. (ii) Surprisingly, the prevalence of p-tau during hibernation was higher among net-bearing neurons (82%) than in the overall neuronal population (60%). Thus, in contrast to the hypothesis that net-bearing neurons are devoid of p-tau, as evidenced by investigations of AD patient brains, PNN-ensheathed neurons were in fact more likely to exhibit p-tau during torpor than the neocortical neurons in their entirety. (iii) We found a strong association between PNNs, p-tau, and PV, with approximately 81% of PV+ net-bearing neurons displaying p-tau during torpor.

Our results demonstrate that the number of net-bearing neurons in relation to the total neuronal population in the MC and PSC remained consistent throughout the hibernation phase: approximately 6% of neurons in the MC and 11% of neurons in the PSC exhibited PNNs in both the euthermic and torpid states. These findings align with prior research reporting that the activity-dependent formation and maturation of PNNs^93,94^ typically occurs over the course of several weeks to months in rodents^95,96^ and even longer in humans^97^ during development. Thus, a drastic increase in PNN expression during the relatively brief periods of euthermia and torpor, which only span a duration of multiple hours and up to 3–4 days, respectively^63,98^, are not to be expected. On the contrary, circadian changes in PNN expression have been demonstrated in brain regions involved in emotional memory processing^99^, indicating that enzymatic degradation of PNNs or PNN components can be completed in a shorter timeframe. Nevertheless, our results concur with previous studies which did not observe alterations in PNN expression in the adult mouse barrel cortex in response to altered sensory activity^21^, and in sensorimotor areas throughout the hibernation cycle of the 13-lined ground squirrel^100^. Furthermore, the maturation of PNNs especially around PV+ interneurons coincides with the closure of critical periods in several sensory systems, possibly by stabilizing synaptic contacts and thereby preserving the acquired state^21,23,101^. Given that hibernating animals undergo drastic changes without apparent brain damage when entering and arousing from torpor^67^, it seems possible that PNNs may serve to maintain established synaptic connections during hibernation, therefore explaining why we did not find any difference in PNN expression between euthermic and torpid animals. However, further research is necessary to determine the proposed role of PNNs in the torpor phase of hibernating organisms.

In addition to their function in restricting neuroplasticity during critical periods, PNNs are also posited to possess neuroprotective properties. For instance, it has repeatedly been shown that net-bearing neurons appear to be devoid of hyperphosphorylated and aggregated tau protein in brains of AD patients, suggesting that PNNs provide protection against pathological forms of tau^33–36^. Phosphorylation of the microtubule-associated protein tau leads to its detachment from the microtubules and translocation into the somatodendritic compartment^48–50^ where it accumulates and, in the case of AD, forms pathological aggregates which possibly promote neurodegeneration^51–53^. However, reversible hyperphosphorylation of tau has been described as a physiological phenomenon during torpor in multiple species, including the Syrian hamster^58,66^, which was utilized in our experiments. In Syrian hamsters, no pathological conformational change of p-tau has been observed during hibernation and the tau protein rapidly reverts to its dephosphorylated state upon arousal, highlighting the hyperphosphorylation of tau as a physiological process during torpor. In light of the main result of the present study, it is important to distinguish between the hyperphosphorylation of tau in pathological and physiological contexts: not only did PNN-ensheathed neurons exhibit p-tau during hibernation, but the prevalence of p-tau among net-bearing neurons (82%) even exceeded that observed in the overall neuronal population (60%) in the analyzed brain regions. Although a complex mutual interrelation of p-tau and PNN components has recently been reported^102^, the functional details of this interplay remain elusive. It has previously been suggested that, in the context of tauopathies, PNNs may act as a barrier to the internalization of extracellularly spread p-tau, rather than preventing intracellular tau hyperphosphorylation in the first place^103,104^. The outcome of this study demonstrates that further research is required to gain insight into the functional details of the relationship between PNNs and p-tau in both pathological and physiological processes.

To approach the question which subset of net-bearing neurons showed a strong correlation with the formation of p-tau during hibernation, we investigated the distribution of the calcium-binding protein parvalbumin among PNN-enwrapped neurons, as PNNs often surround fast-spiking PV+ inhibitory interneurons^14^. Approximately 63% of PNNs enwrapped PV+ interneurons in the MC and 57% in the PSC of euthermic animals. Considering the variance of our results, these findings are largely consistent with those obtained from mice and rats with reported proportions of PV+ interneurons among net-bearing neurons of about 74% in the MC^89^ and 49–75% in the PSC^28,89,92^.

Although no significant difference was observed between the euthermic and torpid states in the present work, the regional variations were more pronounced during torpor, with a markedly reduced number of PV+ interneurons among net-bearing cells in the PSC compared to the MC. Similarly, the expression of PV-like protein in the garden snail (*Cornu aspersum*) was found to remain unaltered throughout the hibernation cycle^90^, while, in the Etruscan shrew (*Suncus etruscus*), the number of PV+ interneurons decreased in the somatosensory cortex during winter^91^. Given that during this period of reduced food availability, energy conservation becomes a priority, the reduction in the number of highly active PV+ interneurons may yield direct metabolic benefits. However, the number of PV+ interneurons among net-bearing cells remained constant during torpor compared to euthermic conditions in the present study. This suggests that subtle yet region-specific alterations in PV turnover may be involved in the complex mechanisms associated with hibernation, where additional adaptations may result in a reduced metabolic state. For instance, the hyperphosphorylation of tau may contribute to the optimization of energy expenditure during torpor. Our data demonstrate that the majority of PV+ net-bearing neurons (81%) exhibited p-tau during hibernation. Furthermore, approximately 78% of PV+ interneurons expressed p-tau, regardless of their PNN-status. These data suggest that the high prevalence of p-tau in the torpid brain is not exclusively associated with the co-expression of PV and PNNs, but rather with the presence of either PNNs or PV. In conclusion, we demonstrated that PV can serve as a marker for subsets of neurons and net-bearing neurons exhibiting a higher prevalence of p-tau than the overall neuronal population. Both PNNs and PV are associated with highly active, fast-firing, and thus energy-expensive neurons^12,18,30,105^, and p-tau has been reported to decrease neuronal activity and excitability^83–85^. Given the virtual cessation of neuronal activity that has been described to occur in cortical and midbrain regions during hibernation^63–65^, we hypothesize that the hyperphosphorylation and subsequent translocation of tau into the somatodendritic compartment may play a significant role in the silencing of energy-expensive cortical neurons during torpor.

The protein tau is known to facilitate the assembly and stabilization of microtubules, thereby participating in the regulation of axonal transport and cytoskeletal remodeling, among other processes^44–47^. By altering these functions, the hyperphosphorylation of tau during torpor may potentially contribute to neuronal silencing via a number of different mechanisms. For instance, p-tau could impair synaptic transmission by decelerating axonal transport of synaptic vesicles to the presynapse as has been previously reported^45,106^. Additionally, p-tau could mediate mitochondrial redistribution from dendrites and presynaptic terminals to perinuclear regions by the same mechanism^107,108^. Such an effect would have significant impact on fast-firing PV+ interneurons, which typically contain a greater number of mitochondria in these compartments compared to other neurons, possibly to fulfill their high energy demands^109,110^. This is in alignment with results of the present study, which demonstrate that the vast majority of (PNN-enwrapped) PV+ inhibitory interneurons exhibited p-tau during torpor.

Furthermore, cytoskeletal restructuring, such as dendritic spine regression and/or modifications to the axon initial segment (AIS) may contribute to reduced neuronal activity by hyperphosphorylation of tau. Prior research demonstrated that dendritic spine morphology changes greatly in the cortex, hippocampus, and thalamus of hibernating species during torpor^111–113^, and the reversible retraction of dendritic spines was correlated with tau phosphorylation^58,114^. An alteration of axo-dendritic contacts may therefore result in decreased postsynaptic transmission which in turn could lead to attenuated neuronal excitability. Moreover, p-tau led to distal relocation of the AIS and a subsequent reduction in neuronal excitability in pathology mouse models^83^, and lengthening of the AIS has been described in p-tau-expressing neurons during hibernation^115^. Given that action potentials are initiated at the AIS, this compartment displays a unique, activity-dependent cytoskeletal organization to modulate neuronal excitability^116,117^. Therefore, by altering the structure of the AIS, p-tau could possibly influence the generation of action potentials and, by extension, neuronal activity.

Our data demonstrate that highly active neurons, such as those ensheathed by PNNs and (PNN-enwrapped) PV+ inhibitory interneurons, are especially prone to tau hyperphosphorylation during torpor. This suggests that the formation of p-tau may be beneficial for these neuronal subsets, presumably due to the proposed silencing effect of p-tau.

PV+ inhibitory interneurons have been demonstrated to play a pivotal role in synchronized network activity, network oscillations, and the regulation of excitation/inhibition (E/I) balance^30,118–120^, as they project to nearly all local pyramidal neurons within their vicinity^121^, enabling them to control microcircuits^122^. Dysfunction of PV+ neurons in AD mouse models generally tends to cause oscillatory changes and hyperexcitability^123^. However, as the hyperphosphorylation of tau during hibernation is a physiological process, further research is required to elucidate whether network oscillations are also altered during torpor through modulation of PV+ cells. It should also be noted that tau hyperphosphorylation during torpor—here demonstrated by AT8-immunoreactivity—is not confined to PV+ interneurons; rather, our data indicate that approximately 60% of the total neuronal population exhibited p-tau. Given that inhibitory neurons in general and PV+ inhibitory interneurons specifically represent only 10–20% and 4–8% of rodent neocortical neurons, respectively^87,88,124–127^, it can be inferred that the majority of p-tau-expressing neurons must be excitatory. Indeed, tau hyperphosphorylation may be a vital mechanism in excitatory neurons to counter the proposed p-tau-mediated downregulation of PV+ inhibitory interneurons and prevent excitotoxicity. Therefore, the physiological hyperphosphorylation of the tau protein during torpor may contribute to the E/I balance that is specifically required for hibernation.

In conclusion, our results reinforce the importance of distinguishing between tau hyperphosphorylation in pathological versus physiological conditions. Specifically, our work revealed that PNN-enwrapped neurons, including PV+ inhibitory interneurons, displayed a high prevalence of p-tau during torpor, contrary to findings in AD pathology, where net-bearing neurons are typically protected from tau pathology. Understanding the mechanisms of the interaction between p-tau, PNNs, and PV+ interneurons could therefore illuminate adaptive strategies for neuronal protection and metabolic regulation in health and disease, offering broader implications for conditions of neurodegeneration and metabolic stress in the brain.

## Data Availability

All of the raw and processed image data, as well as all text-based data (csv files) and scripts that process these data, are available upon request from Valerie Berrouschot (valerie.berrouschot@medizin.uni-leipzig.de) or Markus Morawski (markus.morawski@medizin.uni-leipzig.de).
